# Language input in late infancy scaffolds emergent literacy skills and predicts reading related white matter development

**DOI:** 10.3389/fnhum.2022.922552

**Published:** 2022-11-15

**Authors:** Yael Weiss, Elizabeth Huber, Naja Ferjan Ramírez, Neva M. Corrigan, Vasily L. Yarnykh, Patricia K. Kuhl

**Affiliations:** ^1^Institute for Learning and Brain Sciences, University of Washington, Seattle, WA, United States; ^2^Department of Speech and Hearing Sciences, University of Washington, Seattle, WA, United States; ^3^Department of Linguistics, University of Washington, Seattle, WA, United States; ^4^Department of Radiology, University of Washington, Seattle, WA, United States

**Keywords:** reading development, language development, brain imaging, parental language input, LENA, white matter myelination, longitudinal, conversational turns

## Abstract

Longitudinal studies provide the unique opportunity to test whether early language provides a scaffolding for the acquisition of the ability to read. This study tests the hypothesis that parental language input during the first 2 years of life predicts emergent literacy skills at 5 years of age, and that white matter development observed early in the 3rd year (at 26 months) may help to account for these effects. We collected naturalistic recordings of parent and child language at 6, 10, 14, 18, and 24 months using the Language ENvironment Analysis system (LENA) in a group of typically developing infants. We then examined the relationship between language measures during infancy and follow-up measures of reading related skills at age 5 years, in the same group of participants (*N* = 53). A subset of these children also completed diffusion and quantitative MRI scans at age 2 years (*N* = 20). Within this subgroup, diffusion tractography was used to identify white matter pathways that are considered critical to language and reading development, namely, the arcuate fasciculus (AF), superior and inferior longitudinal fasciculi, and inferior occipital-frontal fasciculus. Quantitative macromolecular proton fraction (MPF) mapping was used to characterize myelin density within these separately defined regions of interest. The longitudinal data were then used to test correlations between early language input and output, white matter measures at age 2 years, and pre-literacy skills at age 5 years. Parental language input, child speech output, and parent-child conversational turns correlated with pre-literacy skills, as well as myelin density estimates within the left arcuate and superior longitudinal fasciculus. Mediation analyses indicated that the left AF accounted for longitudinal relationships between infant home language measures and 5-year letter identification and letter-sound knowledge, suggesting that the left AF myelination at 2 years may serve as a mechanism by which early language experience supports emergent literacy.

## Introduction

Examining whether early language skills predict later emergent literacy skills in preschoolers can shed light on the relationship between language and literacy development and may contribute to developing effective early instruction and interventions and early identification of children at risk of developing reading difficulties. Early language measures coupled with neuroimaging measures in the developing brain can further illuminate the interplay between language input, early language skills, and the emerging neural circuitry for reading. The current study examined the relationship between early language skills and parental language input in infancy (6–24 months), white matter structure at age 2 years, and emergent literacy skills in 5-year-old preschoolers. Additionally, white matter structure at 2 years was tested as a potential mediator of the relationship between infant and preschool behavioral measures.

### Predictors of emergent literacy skills

Phonological awareness and letter-sound knowledge have consistently been found as the two best predictors of reading acquisition during the first 2 years in school (Share, 2004; NRP, 2020). These two skills are necessary to develop decoding, which is the ability to activate speech-based information from a string of printed letters (Share, 1995). However, according to the simple view of reading (SVR) framework (Hoover and Gough, 1990), skilled reading reflects adequate reading comprehension. That is, according to SVR, the ability to develop good reading skills depends on the child’s language comprehension skills and their ability to decode written words. Consequently, the SVR framework hypothesizes that decoding and language comprehension are two distinct components, and that reading comprehension is the product of these two components. The SVR framework further predicts that reading difficulties can result from inadequate decoding skills, inadequate language comprehension skills, or both (Hoover and Gough, 1990).

Longitudinal and cross-sectional studies have supported the SVR framework by demonstrating that concurrent language comprehension and decoding skills strongly predict reading comprehension skills at school age. These studies also extend the SVR framework by showing that language comprehension and decoding skills in preschool, before the onset of formal literacy instruction, predict reading comprehension skills at school age even several years later (Kendeou et al., 2009; Hjetland et al., 2017, 2019; Chiu, 2018; Dickinson et al., 2019). However, while Kendeou et al. (2009) further support the SVR framework by indicating that language and decoding skills are two distinct components, other studies challenge it by demonstrating strong correlations between decoding and language comprehension in preschoolers, indicating that these are not two distinct components and instead depend on one another (Chiu, 2018; Dickinson et al., 2019; Hjetland et al., 2019). These inconsistencies are suggested to be due to the use of different measures and analytical approaches among different studies (Hjetland et al., 2017). For example, Kendeou et al. (2009) used factor analysis and included receptive vocabulary under the decoding skills component, while other studies used structural equation modeling (SEM) or path and mediation analysis and included vocabulary measures under the language comprehension component (Chiu, 2018; Dickinson et al., 2019; Hjetland et al., 2019). Furthermore, while most of these studies include only standardized measures, others include natural measures from language samples as well (for a comprehensive review see Hjetland et al., 2017). These inconsistencies highlight the need to examine the specific relations between spoken language and decoding skills prior to the onset of literacy instruction.

### Language predictors of emergent decoding skills

Despite the clear contribution of language skills to reading comprehension in school-age children, it is not yet clear how early language skills relate to emergent literacy skills. Different studies show inconsistencies related to the specific nature of these relations and suggest that there are additional contributing factors. One comprehensive study explicitly addressed the question regarding the relations between early language skills and emergent and later literacy skills using path analysis (NICHD Early Child Care Research Network, 2005). This study indicates that comprehensive language skills at both 36 and 54 months were directly related to the standardized measures of both phonological awareness and letter and word identification at 54 months. These results indicate both concurrent and longitudinal relations between language and emergent literacy skills in preschoolers.

Other studies have mostly focused on the relations between early vocabulary and emergent literacy skills. For example, Deniz Can et al. (2013) showed that expressive vocabulary (measured by a parental questionnaire) at the age of 2 years predicted emergent literacy skills, including standardized measures of letter-sound correspondence, word recognition, and decoding in kindergarten, but not phonological awareness or letter naming. However, these emergent literacy skills were correlated with concurrent language skills, including standardized measures of vocabulary, syntax, and semantics. Another longitudinal study followed children from 19 months to 16 years (Suggate et al., 2018). They found that vocabulary (measured by a parental questionnaire) at 19 months correlated significantly with emergent literacy skills including standardized measures of letter and word recognition and concept about print, 2–3 years later. In addition, they found that maternal receptive vocabulary (measured by a standardized test) when children were 19-months-old, was related to both early vocabulary and emergent literacy skills in the children. Finally, Silvén et al. (2002) found that both infant’s expressive and receptive language skills and mother’s interactional sensitivity (both measured with an observational approach) at 12 and 24 months predicted standardized measures of emergent phonological awareness skills at 36 and 48 months (Silvén et al., 2002). These two studies highlight the importance of parental input and the home environment in infancy to the development of emergent literacy skills.

There is evidence of long-term predictive associations between early language skills on later reading skills. Flax et al. (2009) found that expressive and receptive language skills (measured with both a standardized test and a parental questionnaire) in the 2nd and 3rd years of life predicted standardized measures of phonological awareness at age 5-years and word identification and decoding at age 7-years. Psyridou et al. (2018) found that receptive and expressive vocabulary (measured by a parental questionnaire) at 24 and 30 months predicted reading comprehension in 2nd, 3rd, 8th, and 9th grades. Duff et al. (2015) found that receptive and expressive vocabulary (measured by a parental questionnaire) in the 2nd year of life predicted different skills at the age of 4–9 years.

### The home language environment

Parental language input is a key component of the early learning environment, and child language development is associated with input quantity, as well as several dimensions of input quality [for a recent review, see Rowe and Snow (2020); Rowe and Weisleder (2020)]. For example, a greater quantity of child-directed speech input has been linked to more advanced expressive vocabulary (Huttenlocher et al., 1991; Hart and Risley, 1995; Hurtado et al., 2008; Shneidman et al., 2013) and stronger lexical processing skills in the 2nd year of life (Weisleder and Fernald, 2013). Linguistic quality of parental speech, including lexical and syntactic diversity, complexity, and narrative content (Huttenlocher et al., 1991, 2002, 2010; Tabors et al., 2001; Pan et al., 2005; Rowe, 2012; Song et al., 2014; Hsu et al., 2017; Uccelli et al., 2019; Leech, 2021) has also been shown to correlate with child language outcomes.

Parental speaking style is another important element of input quality. Adults tend to adopt a style known as “parentese” when they address infants and young children (Fernald, 1985; Fernald and Kuhl, 1987; Grieser and Kuhl, 1988). Parentese is characterized by a slower pace and higher, more variable pitch (Fernald, 1985; Fernald and Kuhl, 1987). Parents have been found to use parentese during activities like book reading (Burnham et al., 2015) as well as during spontaneous child-directed speech. Benefits associated with parentese have been reported for several aspects of language acquisition (Cristià, 2013), including phonemic discrimination (Liu et al., 2003), and vocabulary development (Hartman et al., 2017). For example, Hartman et al. (2017) showed a specific relationship between vowel clarity in maternal parentese at 16 months and children’s receptive and expressive vocabulary size at age 2 years (Hartman et al., 2017). In the laboratory, parentese has been shown to facilitate speech segmentation (Thiessen et al., 2005), as well as word recognition and learning in infancy (Singh et al., 2009; Ma et al., 2011). Exposure to parentese in the home environment has been found to correlate with child language output, including speech-like “babbling” in infancy (Ramírez-Esparza et al., 2014; Ferjan Ramírez et al., 2019).

Social interaction is also thought to play an important role in language acquisition, and is considered another important dimension of input quality (Rowe and Snow, 2020; Rowe and Weisleder, 2020). Studies that manipulate social-interactional variables, through intervention or experimental methods, suggest that interaction with responsive adults directly advances infant and child language skills (Kuhl et al., 2003; Goldstein and Schwade, 2008; Ferjan Ramírez et al., 2019, 2020). In correlational studies, conversational turn counts have been associated with immediate as well as longer-term linguistic and cognitive outcomes (Gilkerson et al., 2017, 2018). Moreover, there is evidence that the quantity of parent-child conversational turns correlates not only with child linguistic and cognitive performance but also with measures of brain function and structure (at 4–6 years of age) (Romeo et al., 2018a,b, 2021). In particular, conversational turns counts have been found to correlate with the organization of dorsal white matter pathways associated with expressive and receptive language skills in both children and adults (Romeo et al., 2018b).

The early language environment has also been linked to specific components of child literacy skills. Parent-child book reading interactions at 1–2.5 years have been shown to predict receptive vocabulary, reading comprehension, and internal motivation to read, but not decoding, external motivation to read, or math skills at elementary school (2nd–4th grade) (Demir-Lira et al., 2019). Furthermore, studies that measured the quality of the learning environment, including literacy activities, quality of maternal engagement, and availability of learning materials during the 2nd and 3rd years of life, found that Preschool receptive vocabulary and letter-word identification skills were associated with the quality of the learning environment in the 2nd and 3rd years, as well as the preschool learning environment (Rodriguez and Tamis-Lemonda, 2011; Tamis-LeMonda et al., 2019).

### Brain networks supporting language and early literacy development

Brain imaging studies have shown that reading involves two main brain systems in the left hemisphere. The dorsal sub-lexical pathways maps between print and sounds of spoken language and include temporoparietal and frontal regions and the white matter pathways that connect them, namely the arcuate fasciculus (AF) and superior longitudinal fasciculus (SLF). This system is more involved in the beginning stages of reading development. The dorsal pathways are also associated with speech production and auditory-motor integration (Hickok and Poeppel, 2007; Hickok, 2012; Skeide and Friederici, 2016), as well as phonological awareness, vocabulary development, and syntactic processing of speech (Lebel and Beaulieu, 2009; Saygin et al., 2013; Skeide et al., 2016; Su et al., 2018; Reynolds et al., 2019). The ventral lexical system maps between print and meaning and includes occipital and occipitotemporal regions and the inferior fronto-occipital and inferior longitudinal fasciculus (IFOF and ILF), which connect them. This system becomes more automatic with reading experience (Wandell and Yeatman, 2013; Ozernov-Palchik and Gabrieli, 2018; Church et al., 2021).

Longitudinal studies measuring brain function in children from the pre-reading stage throughout reading development have shown that activation in the left posterior superior temporal gyrus and functional connectivity between the left dorsal and ventral pathways, which is related to phonological processing in pre-readers, predict reading skills 1–3 years later at school-age (Yu et al., 2018; Wang et al., 2020; Yamasaki et al., 2021). Similarly, longitudinal studies that measured brain structure in children from the pre-reading stage throughout reading development consistently demonstrate that throughout development the left AF relates to phonological awareness skills that are essential for reading acquisition (Lebel and Beaulieu, 2009; Yeatman et al., 2011; Saygin et al., 2013; Van Der Auwera et al., 2021). Other studies have demonstrated correlations between left AF and cross-modal audio-visual processing in school-age children, and that the direct segment of the AF specifically predicts later reading skills (Gullick and Booth, 2014, 2015). Altogether, these longitudinal studies indicate a strong correlation between the left dorsal white matter and function and reading-related language skills, even before the onset of reading instruction.

Studies with infants (3–12 months) and young children (1–5 years) suggest that white matter development coincides with the emergence of language-related skills. For example, estimates of left AF and SLF fractional anisotropy (FA) obtained shortly after birth have been shown to correlate with receptive and expressive language skills at the age of 2 years (Salvan et al., 2017; Girault et al., 2019a; Sket et al., 2019). Furthermore, magnetic resonance imaging (MRI) myelin water fraction estimates have been shown to correlate with early linguistic and cognitive skills, through 5 years of age (O’Muircheartaigh et al., 2014; Deoni et al., 2016). Similarly, changes in FA from 6 to 24 months have also been found to predict expressive language skills at 24 months (Swanson et al., 2017), suggesting that white matter development during this period influences subsequent language skills. However, it is yet unknown whether the brain structure earlier in life is related to and mediates the correlations between early language, environmental factors, and later reading outcomes.

### The current study

In the current study, we examined how infants’ spoken language environments and early language skills relate to emergent literacy skills at the age of 5 years. Based the research described above, we hypothesized that parental input and parent-child interactions in the first 2 years of life would predict later pre-reading skills, and that this relationship might be mediated by structural development of the white matter, specifically within pathways related to expressive language skills. Longitudinal data included: (1) Measures of infants’ spoken language, parental input, and interactions with caregivers from 6- to 24-months of age, manually coded from home language recordings; (2) Diffusion and quantitative MRI at 26 months; (3) Emergent literacy and related skills at 5-years of age. Correlation and regression analyses were used to assess the relationship between early spoken language skills and parental input, decoding skills prior to the onset of literacy instruction, and the brain mechanisms that support these relations.

## Materials and methods

### Participants

Seventy-nine families from the Seattle metro area were recruited through the University of Washington (UW) Communications Studies Participants registry with UW Human Subjects Approval that provides subject contact information directly to researchers. These families had previously participated in intervention studies at the Institute for Learning and Brain Sciences (I-LABS) (Ferjan Ramírez et al., 2019, 2020, 2021) and agreed to be re-contacted for future research on their consent forms. All experimental procedures were approved by the UW Institutional Review Board, and all participating families gave informed consent and were compensated monetarily for their time and effort. All families completed audio recordings of their children and environment at five time points in infancy (when infants were 6, 10, 14, 18, and 24 months old). The recordings were employed with the widely used Language ENvironment Analysis system (LENA™ Pro Version 3.4.0, LENA, 2015), which provides audio recordings and measures of different components in children’s natural environments (For a more detailed description of the data collection and analysis of the LENA recordings at infancy, see Ferjan Ramírez et al., 2019, 2020, 2021).

As noted above, some families (*N* = 38 total, and *N* = 16 in the MRI group) participated in a parental language intervention from child ages 6–18 months. The intervention increased the quantity of parentese speech and parent-child conversational turns observed in home language recordings (Ferjan Ramírez et al., 2019, 2020, 2021), as well as expressive language outcomes from 14 to 30 months of age (Ferjan Ramírez et al., 2019, 2020). The goal of the current study was to evaluate the effects of early language experience, rather than the effects of this specific intervention, which have been described previously. Therefore, data were analyzed across all participants, regardless of their participation in the intervention program. It should be noted, however, that the current sample likely has greater variability and higher rates of parentese speech and parent-child interaction than would be expected in a comparable, non-intervention sample.

All families who participated in the study when their children were infants were invited to return for a follow-up MRI session when children were approximately 26 months old, as well as a follow-up study of pre-reading skills at the age of 5 years. All families who agreed to have their child participate in a follow-up study at the age of 5 years (*N* = 70) completed an initial phone screening interview to determine whether their children met the following criteria: (1) Pre-K child between the age of 5 years and 5 years and 4 months; (2) Native English is primary in the home (multi-lingual families were included if English was spoken >65% of the time in the home, based on parental report during pre-enrollment screening); (3) Children had no clinically diagnosed congenital, neurological or other physical abnormality. Exclusion criteria included: (1) Any brain injury and medications that impact cognition; (2) Intellectual disability, Autism Spectrum Disorder, mood disorders, and other disorders that impact cognition; (3) Significant and permanent hearing impairments. After the initial screening process, 53 eligible participants were invited to take part in the follow-up study at I-LABS when they were 5-years-old (Weiss et al., 2022). Among the 53 participants who participated in the follow-up study at the age of 5 years, a smaller group also completed brain imaging data collection at 26 months (*N* = 20, 12 females, *M* = 27.34 months, SD = 0.73).

### Procedure

#### Measures at 5 years of age

All 53 eligible participants’ families completed an online parental questionnaire that included questions regarding children’s health and development history, language learning history, family history of dyslexia and reading difficulties, parental education, and socio-economic status (SES). Participants’ reading and related skills at the age of 5-years were measured with the following standardized and non-standardized tests that were adapted to online administration by uploading the stimuli to PowerPoint presentations and presented to the participants during online sessions via Zoom (Weiss et al., 2022). The online administration of these tests was a response to the COVID-19 pandemic, when it was not possible to meet with participants in person. Online tests were only used to assess participant’s relative performance level, and not as the basis for any kind of formal diagnosis. All participants went through the same procedures, which are also reported in a previous publication (Weiss et al., 2022).

##### Letter knowledge test

This test is designed to measure Alphabet knowledge and letter sounds. Participants were shown isolated letters on the screen and instructed to name the letters and their corresponding sounds. This test was administered separately for lowercase and uppercase letters. This test resulted in four measures: Uppercase letter names (ULN), uppercase letter sounds (ULS), lowercase letter names (LLN), and lowercase letter sounds (LLS). All 26 letters were presented in random order.

Letter sound scoring was carried out by trained research assistants, under the supervision of the first author. Scorers only accepted isolated pronunciation (not adding any vowel), short vowels, and hard “G,” “C,” and “X” as correct responses. Video recordings of each session were first scored by the research assistant who administered the session. Two other research team members then provided independent scoring for the videos. Inter-rater inconsistencies were discussed in weekly meetings and resolved as a group.

##### Woodcock reading mastery tests-third edition

This standardized test (Woodcock, 2011) is designed to assess reading skills in children and adults. We administered the Phonological Awareness (PA) sub-test. Different versions (forms A and B) were counterbalanced between participants.

##### Expressive vocabulary test-third edition

This standardized test (Williams, 2018) is designed to assess expressive vocabulary test (EVT) and word retrieval based on words in Standard American English in children and adults. Different versions (forms A and B) were counterbalanced between participants.

#### Language environment and child language measures from 6 to 24 months of age

The Language ENvironment Analysis System (LENA™ Pro Version 3.4.0, LENA, 2015) was used to collect naturalistic first-person recordings from all families over two weekend days when children were 6-, 10-, 14-, 18-, and 24-months-old. Each recording was first pre-processed with the LENA Advanced Data Extractor Tool (ADEX). Custom software was used to identify 50 unique 30-s intervals per day containing the highest daily adult word count (AWC), spaced at least 3 min apart. As described previously (Ferjan Ramírez et al., 2019, 2020), this step identifies intervals with enough language data for analysis and eliminates uninformative periods (e.g., nap times). A total of 100 30-s intervals (50 intervals per day) were obtained for each participant at each age.

Measures of parental language input and child output were then manually coded from the LENA recordings by trained research assistants, following procedures outlined previously (Ramírez-Esparza et al., 2014, 2017a,2017b; Ferjan Ramírez et al., 2019, 2020, 2021). Coders tabulated the number of intervals containing parentese speech (PT) and/or child speech or speech-like vocalizations (CS), and the total number of parent-child conversational turns (CT) present within each 30-s interval. Ten individuals performed coding for each language variable, and inter-coder reliability was assessed using methods developed by Ramírez-Esparza et al. (2014). Intraclass correlation coefficients (ICC) indicated a high degree of inter-coder agreement (see also Shrout and Fleiss, 1979; Ramírez-Esparza et al., 2017a). ICC estimates for PT, CS, and CT were 0.95, 0.93, and 0.96, respectively.

Although the LENA software package can be used to obtain automated counts of adult words, child vocalizations, and parent-child conversational turns, recent validation studies have shown that these automated estimates are susceptible to error and bias, especially for conversational turn counts in the age range studied here (6–24 months) (Bulgarelli and Bergelson, 2020; Cristià et al., 2020, 2021; Ferjan Ramírez et al., 2021), due to factors like accidental contiguity between speakers (e.g., parent speaking on the phone while the child is babbling to herself, nearby), sibling speech, and noise in the recordings. We, therefore, focused our analysis on manually coded variables. Exact variable definitions, provided in Table 1, were based on criteria previously established in the literature (Ramírez-Esparza et al., 2014, 2017a,2017b; Ferjan Ramírez et al., 2019, 2020, 2021).

**TABLE 1:** Parent and child language variables measured from 6 to 24 months.

Variable name	Variable definition
Parentese (PT)	Total intervals in which mother, father, or another adult spoke to the infant using parentese speech (high pitch, slow tempo, and exaggerated contours), either alone or in the presence of other adult voices
Child speech and speech-like vocalizations (CS)	Total intervals in which children either repeated or independently produced one or more of the following: fully resonant vowels, consonant–vowel syllables, syllable strings, speech utterances intermixed with non-speech, word-like strings, single words, or word strings
Conversational turns (CT)	Total number of adult utterances directed to child followed within 5 s by a child utterance directed to adult, or vice versa; counted in discrete pairs (child-to-parent = 1 turn, parent-to-child-to-parent = 1 turn, child-to-parent-to-child-to-parent = 2 turns)

Variable definitions used in manual coding of the Language Environment Analysis System (LENA) recordings at each age.

#### Magnetic resonance imaging acquisition at the age 2 years

All data were acquired using a 3.0 T Philips Ingenia MRI system with a 32-channel head coil while children were in natural sleep. High resolution T1-weighted images were acquired using a multi-echo MPRAGE sequence with FOV = 230 × 230 × 180, acquisition voxel size 1.0 mm × 1.0 mm × 1.0 mm, reconstructed voxel size 0.5 mm × 0.5 mm × 0.5 mm, TR/TI/TE1/TE2 = 13/1200/3.7/9.7 ms, shot interval 2,250 ms, and flip angle (FA) = 8°. T1-weighted images were used as a common reference space for later anatomically guided analysis of macromolecular proton fraction (MPF) maps and of diffusion-weighted images, as described below.

For MPF mapping, a fast 3D protocol was implemented according to the single-point synthetic reference method (Yarnykh, 2012, 2016), which included three spoiled gradient-echo sequences with magnetization transfer (MT) (TR = 31 ms, FA = 8°), proton-density (TR = 21 ms, FA = 4°), and T1 (TR = 21 ms, FA = 25°) contrast weightings. Off-resonance saturation in the MT-weighted sequence was applied at the offset frequency 4 kHz with effective FA = 430° and pulse duration 7 ms. All images were obtained in the sagittal plane with dual-echo readout (TE1/TE2 = 4.9 ms/10.0 ms), FOV = 240 × 240 × 200 mm^3^, and actual voxel size of 1.25 mm × 1.25 mm × 1.24 mm interpolated to 0.625 mm × 0.625 mm × 0.620 mm. Additionally, actual flip-angle imaging (AFI) B1 maps (Yarnykh, 2007) (TR1/TR2/TE = 60/240/4.8 ms, FA = 60°, voxel size 2.5 mm × 5.0 mm × 5.0 mm) were acquired in the same geometry and reconstructed with 0.625 mm × 0.625 mm × 0.620 mm voxel size.

Diffusion-weighted data were acquired using a single-shot DWI-EPI sequence with FOV = 230 × 230 × 146, acquisition voxel size 1.8 mm × 1.8 mm × 1.9 mm, reconstructed voxel size 1.4 mm × 1.4 mm × 1.9 mm, TR/TE = 11,926/97 ms, FA = 90°. Each diffusion scan included 6 non-diffusion-weighted (*b* = 0) volumes and 64 diffusion-weighted volumes acquired at either a *b*-value of 2,000 s/mm^2^ (52 non-collinear gradient directions) or a *b*-value of 800 s/mm^2^ (12 additional non-collinear gradient directions). An additional set of 6 non-diffusion-weighted volumes were acquired using the same parameters with a reversed phase encoding direction (posterior–anterior), for use in correcting EPI distortions (Andersson et al., 2003), as described below.

##### Macromolecular proton fraction mapping

Macromolecular proton fraction maps were reconstructed according to a single-point synthetic reference algorithm (Yarnykh, 2016) with correction of B1 field non-uniformity using custom-written C-language software with previously determined constraints for the non-adjustable two-pool model parameters (Yarnykh, 2012). Software for reconstruction of MPF maps is available at https://www.macromolecularmri.org. Correction of B0 field inhomogeneity was not applied because of a negligible effect of B0-related errors on MPF measurements (Yarnykh et al., 2020). Prior to map reconstruction, individual echo images in each data set were averaged to increase SNR (Helms and Dechent, 2009). Rigid-body registration of the component image volumes was performed using the FLIRT toolbox of the FSL software package (Smith, 2002). Resulting MPF maps were then aligned to each subject’s own T1 weighted anatomical image using rigid body registration.

Macromolecular proton fraction is sensitive to changes in myelin content in both gray and white matter (Corrigan et al., 2021), and histological validation studies support a linear relationship between MPF and relative myelin density (Underhill et al., 2011).

##### Diffusion magnetic resonance imaging analysis

Diffusion data pre-processing was carried out using the FSL tools (version 6.0.0) for motion and eddy current correction (FSL eddy, Andersson and Sotiropoulos, 2016) and brain extraction (FSL BET, Smith, 2002). Diffusion-weighted volumes first were aligned to an average of the non-diffusion weighted volumes in each scan using rigid body transformation [SPM version 12 (Ashburner and Friston, 1997)]. Volumes were then aligned to the subject’s own T1 weighted anatomical image, again using rigid body registration. Diffusion gradients were adjusted to account for rotation applied during motion correction and registration (Leemans and Jones, 2009).

Whole brain tractography was carried out using the MRtrix software package (version 3.0) with the iFOD2 algorithm (Tournier et al., 2010). The resulting whole brain fiber estimates were then entered into the Automated Fiber Quantification software package (AFQ, Yeatman et al., 2012; software available at https://github.com/yeatmanlab/AFQ). Specifically, initial segmentations were made using way-point ROIs in subject native space. The Mori et al. (2006) atlas was then used to refine these segmentations by removing streamlines with 0–3% likelihood of overlapping the desired anatomy.

##### Tract specific macromolecular proton fraction profiles

Macromolecular proton fraction values were extracted within each fiber tract and summarized at the tract core as a weighted-mean across fiber nodes, which penalizes locations farthest from the spatial center of each tract and thereby minimizes the influence of minor variation in the exact outer boundary of each tract (Yeatman et al., 2012).

Analysis along individual tract locations was carried out by first sampling along 100 evenly spaced locations in native subject space (Yeatman et al., 2012) and then averaging across groups of five nodes to obtain 20 summary locations per subject and tract. Output from AFQ was transformed such that all tract profiles were oriented with nodes increasing (from 0 to 100) right to left, posterior to anterior, and inferior to superior, to simplify presentation of tract profiles. Results were corrected for multiple comparisons along each tract using a permutation-based approach (Nichols and Holmes, 2002), which accounts for spatial similarity within individual white matter tracts (see also Yeatman et al., 2012).

Two participants completed the MPF mapping scans, but not the dMRI scan. For these individuals, MPF profiles were defined as follows: MPF maps were first co-registered to a standard-space template brain, and probabilistic white matter labels were used to define initial candidate tracts (Mori et al., 2006). Tract locations were then visually confirmed in subject native space, relative to the same waypoint ROIs used above, in AFQ (Yeatman et al., 2012). The core of each tract was defined in 3D coordinates using the MATLAB Image Processing Toolbox (using the bwmorph3 and bwconncomp functions), and linearly sampled between termination points at the gray/white matter boundary. All analyses were replicated with and without these participants, to ensure that variation in the definition of fiber bundle “core” values did not change the results.

### Data analysis

#### Behavioral data analysis

First, we wanted to examine whether early parental input and infants’ output measures of natural language environment, as recorded with the LENA system and manually coded in infancy are related to later emergent literacy and related skills at the age of 5 years. For the total sample of 53 participants, we calculated the simple bivariate correlations between their early LENA measures at the age of 6, 10, 14, 18, and 24 months, and their emergent literacy and related skills at the age of 5-years.

Second, we examined the correlations between the early LENA measures and 5-years measures for the smaller sub-group of participants that had brain imaging data at 26-months of age (*N* = 20).

In both samples, the average parental education in years was roughly equivalent to a 4-year college degree, with a wide range extending from elementary to postgraduate level degree completion. For both samples, the range of income-to-need ratio (defined as a family’s total annual income divided by its corresponding poverty threshold) included families at or below the federal poverty line (ratio < 1) as well as families ranging well into the upper quadrants of wealth (e.g., ratio = 19.62). The summary statistics of the participants’ gender, age, and SES in each sample are presented in Table 2.

**TABLE 2:** Demographic information of the total and smaller samples.

		All participants (*N* = 53 total)	MRI participants (*N* = 20 total)
Gender	Identify as boys	25 (47%)	7 (35%)
	Identify as girls	26 (49%)	11 (55%)
	Other/Prefer not to answer	2 (4%)	2 (10%)
Age at first session	Mean age in months (SD)	60.82 (0.88)	60.54 (0.74)
Socio-economic status	Average years of parental education (SD)	17.43 (2.07)	16.83 (1.64)
	Income-to-need ratio (SD)	6.46 (3.97)	4.76 (3.77)

#### Brain imaging data analysis

**FIGURE 1:**
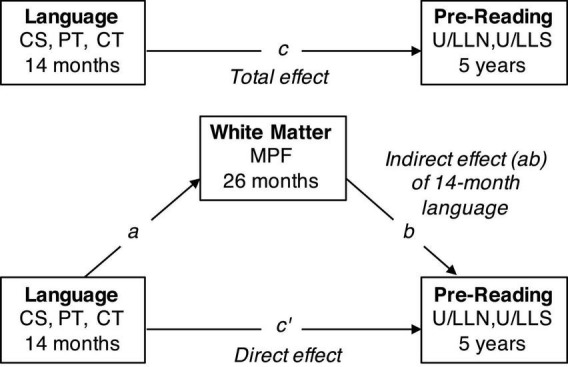
Theoretical mediation model. White matter MPF at 26 months was tested as a hypothetical mediator of the relationship between 14-month language measures [child speech (CS), parentese (PT), and conversational turns (CT)] and 5-year pre-reading skills [upper and lowercase letter name (U/LLN), and upper and lowercase letter sound (U/LLS), knowledge].

Planned comparisons focused on the following white matter tracts: The left and right AF, SLF, inferior longitudinal fasciculus (ILF), and inferior fronto-occipital fasciculus (IFOF). Two regression models were tested within each tract: (1) 26-month MPF values were regressed on each of the LENA language variables of interest (CS, PT, and CT), and (2) pre-reading measures collected at 5 years (ULN, ULS, LLN, LLS, PA, and EVT) were regressed on 26-month MPF values. Average MPF values were then extracted from regions with significant bivariate effects for both infant LENA measures and the 5-year language measures. These values were entered into a mediation analysis designed to test whether 26-month white matter organization accounts for the longitudinal relationship between infant language measures and 5-year pre-reading skills (see Figure 1).

## Results

### Behavioral results

For the whole sample of 53 participants, the simple bivariate Pearson correlations between 6- and 24-months and 5-years of age measures revealed some significant results for both parental input and infants’ output measures. Parental input, as measured by parentese (PT) when infants were 14-months-old is positively correlated with all skills measured at 5-years of age (i.e., uppercase and lowercase letters and sounds knowledge, phonological awareness, and vocabulary) at the age of 5-years. In addition, PT at 6-months and at 18-months old is positively correlated with lowercase letters knowledge at 5-years. However, there are no significant correlations between conversational turn-taking (CT) at 6–24-months-old and the 5-years-old measures.

Infants’ output, as measured by children’s production of speech-like vocalizations (CS) at the age of 14-months old is positively correlated with uppercase and lowercase letter-sound knowledge at 5-years. However, CS at 6-months old is negatively correlated with all 5-years measures. In addition, CS at 24-months old is negatively correlated with uppercase and lowercase letters knowledge. The results are summarized in Tables 3–5.

**TABLE 3:** Correlations between PT (parentese) at 6–24-months and reading-related measures at 5-years of age for the entire sample of 53 participants.

Age	6 months		10 months		14 months		18 months		24 months	
5-years measures	Value	sig.	Value	sig.	Value	sig.	Value	sig.	Value	sig.
ULN	0.023	0.115	0.194	0.185	0.476**†	0.001	0.257	0.081	0.156	0.512
ULS	0.108	0.463	0.081	0.585	0.428**†	0.002	0.204	0.169	0.317	0.174
LLN	0.315*	0.029	0.241	0.099	0.541**†	> 0.001	0.342*	0.018	0.235	0.319
LLS	0.099	0.505	0.069	0.641	0.419**†	0.003	0.191	0.199	0.213	0.366
PA	0.051	0.731	–0.034	0.817	0.328*	0.024	0.008	0.557	0.354	0.126
EVT	0.179	0.223	0.100	0.499	0.381**†	0.008	0.225	0.129	0.285	0.223

All results are calculated for the raw scores for each test. *Significance level < 0.05. **Significance level < 0.01. ^†^Significant after Bonferroni correction for multiple comparisons (*p* ≤ 0.008).

**TABLE 4:** Correlations between CT (conversational turns) at 6–24-months and reading-related measures at 5-years of age for the entire sample of 53 participants.

Age	6 months		10 months		14 months		18 months		24 months	
5-years measures	Value	sig.	Value	sig.	Value	sig.	Value	sig.	Value	sig.
ULN	–0.018	0.902	–0.068	0.646	0.0175	0.233	0.152	0.302	0.239	0.323
ULS	0.089	0.548	0.055	0.708	0.264	0.070	0.158	0.283	0.389	0.099
LLN	0.028	0.848	0.023	0.877	0.232	0.112	0.192	0.190	0.228	0.348
LLS	0.082	0.581	0.050	0.737	0.269	0.064	0.172	0.242	0.317	0.187
PA	0.104	0.483	–0.037	0.804	0.260	0.074	0.054	0.714	0.188	0.442
EVT	–0.044	0.769	–0.141	0.340	0.235	0.108	0.079	0.595	–0.010	0.966

All results are calculated for the raw scores for each test.

**TABLE 5:** Correlations between CS (child speech and speech-like vocalizations) at 6–24-months and reading-related measures at 5-years of age for the entire sample of 53 participants.

Age	6 months		10 months		14 months		18 months		24 months	
5-years measures	Value	sig.	Value	sig.	Value	sig.	Value	sig.	Value	sig.
ULN	−0.329*	0.023	–0.101	0.493	0.103	0.487	–0.020	0.896	−0.657**†	0.002
ULS	−0.436**†	0.002	0.015	0.919	0.309*	0.032	0.011	0.941	–0.359	0.121
LLN	−0.391**†	0.006	–0.074	0.618	0.249	0.087	–0.044	0.769	−0.603**†	0.005
LLS	−0.399**†	0.005	–0.034	0.817	0.347*	0.016	0.077	0.606	–0.389	0.090
PA	−0.288*	0.047	0.030	0.838	0.198	0.177	–0.048	0.750	–0.181	0.445
EVT	−0.388**†	0.006	0.032	0.830	0.092	0.535	–0.042	0.780	–0.387	0.092

All results are calculated for the raw scores for each test. *Significance level < 0.05. **Significance level < 0.01. ^†^Significant after Bonferroni correction for multiple comparisons (*p* ≤ 0.008).

When examining the behavioral results for the sub-group of 20 participants that had also brain imaging data at the age of 26-months, the simple bivariate Pearson correlations between 14-months and 5-years of age measures revealed significant results for both parental input and infants’ output measures. As found for the larger sample, PT at 14-months is positively correlated with uppercase and lowercase letters and sounds knowledge at the age of 5-years. However, it is not significantly correlated with phonological awareness, and vocabulary. PT at 6, 10, 18, and 24 months is not significantly correlated with any 5-years measures. Similarly, as found in the larger sample, CS at 14-months is positively correlated with uppercase and lowercase letter-sound knowledge at 5-years. In addition, CS at 24-months old is negatively correlated with uppercase and lowercase letters knowledge, as found in the bigger sample. In contrast to the larger sample, we also found a significant correlation between CT at 14-months-old and ULS knowledge, and between CT at 6-months and ULS and LLS knowledge (but not with other 5-years-old measures). The results are summarized in Tables 6–8.

**TABLE 6:** Correlations between PT (parentese) at 6–24-months and reading-related measures at 5-years of age for the smaller sample of 20 participants with brain imaging data at 26-months.

Age	6 months		10 months		14 months		18 months		24 months	
5-years measures	Value	sig.	Value	sig.	Value	sig.	Value	sig.	Value	sig.
ULN	0.086	0.720	–0.023	0.923	0.465*	0.039	0.103	0.666	0.156	0.512
ULS	0.106	0.658	0.213	0.336	0.668**†	0.001	0.279	0.223	0.317	0.174
LLN	0.194	0.412	0.075	0.753	0.590**†	0.006	0.290	0.214	0.235	0.319
LLS	–0.049	0.837	0.090	0.705	0.553*	0.011	0.163	0.492	0.213	0.366
PA	–0.128	0.590	–0.084	0.725	0.290	0.215	0.024	0.921	0.354	0.126
EVT	–0.153	0.521	–0.293	0.210	0.228	0.334	–0.119	0.618	0.285	0.223

All results are calculated for the raw scores for each test. *Significance level < 0.05. **Significance level < 0.01. ^†^Significant after Bonferroni correction for multiple comparisons (*p* ≤ 0.008).

**TABLE 7:** Correlations between CT (conversational turns) at 6–24-months and reading-related measures at 5-years of age for the smaller sample of 20 participants with brain imaging data at 26-months.

Age	6 months		10 months		14 months		18 months		24 months	
5-years measures	Value	sig.	Value	sig.	Value	sig.	Value	sig.	Value	sig.
ULN	0.131	0.594	–0.073	0.765	0.310	0.196	0.221	0.363	0.239	0.323
ULS	0.483*	0.036	0.228	0.348	0.473*	0.041	0.415	0.078	0.389	0.099
LLN	0.259	0.284	0.011	0.964	0.341	0.153	0.248	0.306	0.228	0.348
LLS	0.476*	0.039	0.192	0.432	0.409	0.082	0.416	0.077	0.317	0.187
PA	0.025	0.920	–0.171	0.484	0.285	0.236	0.078	0.752	0.188	0.442
EVT	–0.116	0.635	–0.286	0.235	0.242	0.319	–0.100	0.684	–0.010	0.966

All results are calculated for the raw scores for each test. *Significance level < 0.05.

**TABLE 8:** Correlations between CS (child speech and speech-like vocalizations) at 6–24-months and reading-related measures at 5-years of age for the smaller sample of 20 participants with brain imaging data at 26-months.

Age	6 months		10 months		14 months		18 months		24 months	
5-years measures	Value	sig.	Value	sig.	Value	sig.	Value	sig.	Value	sig.
ULN	–0.259	0.269	–0.024	0.920	0.350	0.130	0.024	0.922	−0.657**†	0.002
ULS	–0.400	0.080	0.443	0.051	0.517*	0.020	0.135	0.569	–0.359	0.121
LLN	–0.381	0.097	0.035	0.883	0.430	0.058	–0.066	0.784	−0.603**†	0.005
LLS	–0.264	0.261	0.450*	0.046	0.535*	0.015	0.227	0.336	–0.389	0.090
PA	–0.169	0.477	0.112	0.639	0.148	0.533	0.001	0.997	–0.181	0.445
EVT	–0.0141	0.552	0.121	0.610	0.147	0.536	–0.105	0.659	–0.387	0.092

All results are calculated for the raw scores for each test. *Significance level < 0.05. **Significance level < 0.01. ^†^Significant after Bonferroni correction for multiple comparisons (*p* ≤ 0.008).

### Brain imaging results

**FIGURE 2:**
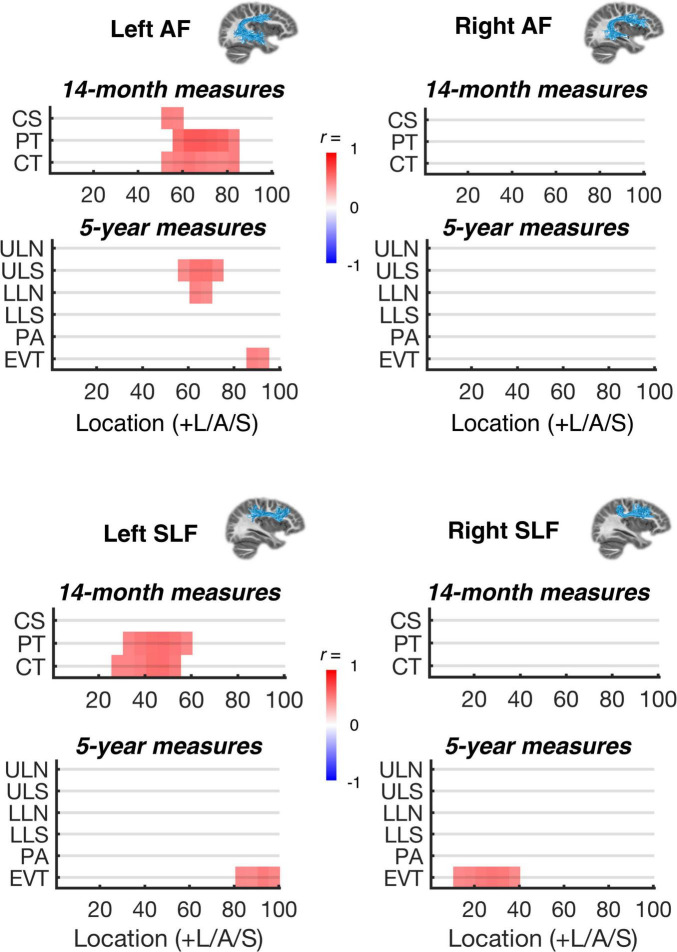
Correlations between language measures and MPF estimates within dorsal white matter pathways. Significant bivariate correlations (*p* < 0.05, corrected for multiple comparisons along each tract; see section “Materials and methods”) are shown for each sampled white matter location within the left and right arcuate and superior longitudinal fasciculus (AF and SLF) and each behavioral variable: 14-month child speech (CS), parentese (PT), and conversational turns (CT); 5-year uppercase letter naming (ULN), uppercase letter sound knowledge (ULS), lowercase letter naming (LLN), lowercase letter sound knowledge (LLS), phonological awareness (PA), and expressive vocabulary test (EVT). Insets (top and middle right) show example tractography-based reconstructions for each of the white matter regions of interest.

**FIGURE 3:**
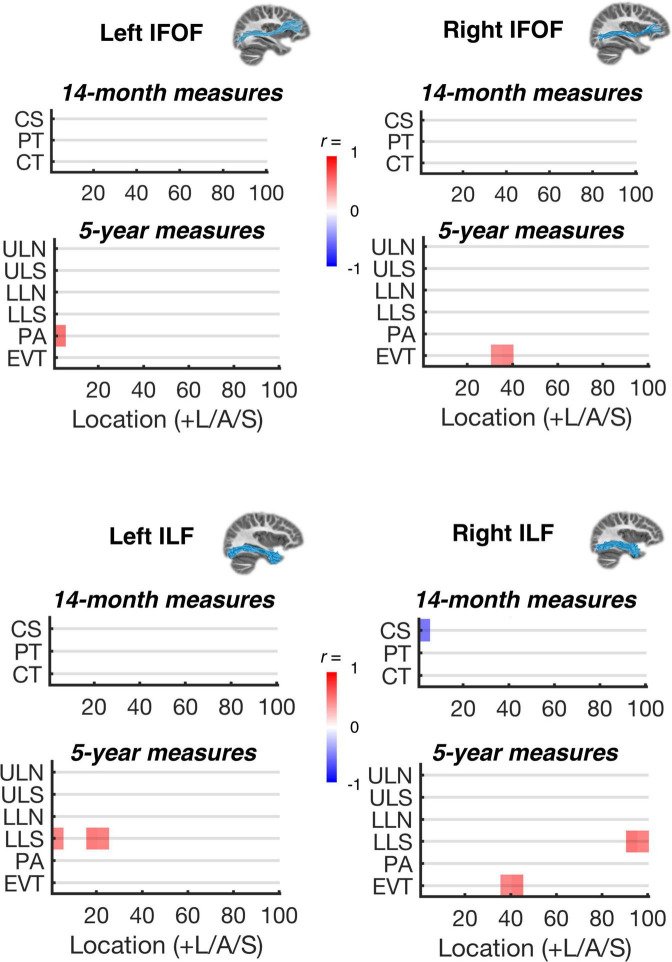
Correlations between language measures and MPF estimates within ventral white matter pathways. Significant bivariate correlations (*p* < 0.05, corrected for multiple comparisons along each tract; see section “Materials and methods”) are shown for each sampled white matter location within the left and right inferior-fronto-occipital and inferior longitudinal fasciculus (IFOF and ILF) and each behavioral variable: 14-month child speech (CS), parentese (PT), and conversational turns (CT); 5-year uppercase letter naming (ULN), uppercase letter sound knowledge (ULS), lowercase letter naming (LLN), lowercase letter sound knowledge (LLS), phonological awareness (PA), and expressive vocabulary test (EVT). Insets (top and middle right) show example tractography-based reconstructions for each of the white matter regions of interest.

For the subgroup of 20 participants with MRI data at 26 months, significant bivariate correlations (2-tailed test; all significant results in the positive direction) were observed within the left AF and left SLF for PT and CT at 14 months, within the left AF for CS at 14 months, and within the left AF for LLN and ULS at 5 years (Figure 2). The significant effects within the left AF showed considerable spatial overlap across the 14-month and 5-year measures. Specifically, the Dice coefficients (Dice, 1945) for PT vs. ULS and LLN were 0.29 and 0.50, respectively. Dice coefficients for CT vs. ULS and LLN were 0.25 and 0.44, respectively. As shown in Figure 3, significant bivariate correlations were also observed for LLS in the left and right ILF, for PA in the left IFOF, and for EVT in the left AF, left and right SLF, right ILF, and right IFOF. However, the effects associated with LLS, PS, and EVT did not co-localize with 14-month CS, PT, or CT, and were less anatomically specific than effects observed for ULS and LLN.

To further assess the relationship between 14-month language measures, 26-months MPF, and 5-years pre-reading measures (ULS and LLN), average MPF values were extracted from regions with significant bivariate effects at both 14 months and 5 years (all within the left AF). Mean MPF values were then tested as mediators for each significant correlation between 14-months and 5-years behavioral measures. As shown in Tables 9, 10, the mediation analyses indicated that 26-months MPF values accounted for 19.73% of the total relationship between CS and ULS (indirect/total effect = 0.1973), and 29.65% of the total relationship between CT and ULS (indirect/total effect = 0.2965). Similarly, 26-month MPF values accounted for 14.92% of the total relationship between PT and LLN (indirect/total effect = 0.1492). In other words, the 26-months MPF values accounted for a significant portion of the variance shared between 14-months CT and 5-years ULS, 14-months CS and 5-years ULS, and 14-months PT and 5-years LLN, consistent with a mediation of these effects by the left AF. Note that CS and CT are not included in Table 10, because the 14-months CS correlations in the white matter did not co-localize with 5-years correlations, and neither of the 14-months CT or CS were significant predictors of 5-years LLN in the direct behavioral correlations.

**TABLE 9:** Mediation analysis for uppercase letter sound knowledge (ULS).

	Total effect (c)		Direct effect (c’)		Indirect effect (ab)	
14-month measures	Estimate	95% CI	Estimate	95% CI	Estimate	95% CI
PT	0.668**	[0.300, 1.037]	0.643*	[0.143, 1.144]	0.025	[−0.002, 0.052]
CS	0.517*	[0.094, 0.941]	0.415	[−0.071, 0.902]	0.102*	[0.078, 0.126]
CT	0.479*	[0.023, 0.936]	0.337	[−0.188, 0.863]	0.142*	[0.110, 0.175]

*Significance level < 0.05. **Significance level < 0.01.

**TABLE 10:** Mediation analysis for lowercase letter knowledge (LLN).

	Total effect (c)		Direct effect (c’)		Indirect effect (ab)	
14-month measures	Estimate	95% CI	Estimate	95% CI	Estimate	95% CI
PT	0.590**	[0.190, 0.990]	0.501	[−0.038, 1.041]	0.088*	[0.060, 0.117]

*Significance level < 0.05. **Significance level < 0.01.

## Discussion

The goal of the current study was to examine how parent and child language variables measured in infancy relate to later emergent literacy skills, and whether white matter development mediates these relationships. We first examined correlations between early parental input and child output measures in the natural language environment from 6 to 24 months, and emergent literacy skills at the age of 5 years. We then examined correlations between quantitative MPF estimates of white matter myelination at 26 months and the longitudinal behavioral measures. Finally, we tested whether MPF values at 26 months mediated the relationships between early language measures and later literacy skills. Parental input and infants’ speech and speech-like vocalizations were found to predict emergent literacy skills at 5-years of age. Furthermore, myelin density estimates in the left AF were found to mediate the correlations between the early language measures and later emergent literacy skills. Together, these longitudinal results add to the literature relating to the long-term effect of early language skills and parental input and suggest that parental input and parent-infant interactions support the development of emergent literacy skills partly through myelination of the left arcuate pathway.

### Correlations between early language input, child output, and emergent literacy skills

Previous studies have demonstrated that expressive and receptive language skills measured in the first 3 years of life predict literacy skills in kindergarten and elementary school, and emergent literacy skills in preschool (Silvén et al., 2002; NICHD Early Child Care Research Network, 2005; Flax et al., 2009; Deniz Can et al., 2013; Duff et al., 2015; Psyridou et al., 2018; Suggate et al., 2018). These relations have been explained by different models and theories. According to the lexical restructuring hypothesis (Metsala and Walley, 2009), vocabulary growth increases phonological awareness of smaller units, which in turn, drives decoding skills. Alternatively, according to the lexical quality hypothesis (Perfetti, 2007) and the triangle model (Seidenberg and McClelland, 1989), better semantic representations contribute to word identification. Our results extend prior findings by linking language experience and behavior in later infancy to specific pre-reading skills, which provide a foundation for early literacy.

Parentese at 14-months of age predicted emergent literacy skills at 5 years of age. Specifically, all of the 5-year measures, including letter names and sound knowledge, phonological awareness, and expressive vocabulary, in the full sample, and letter name and sound knowledge in the smaller sample of participants who had MRI data. These results are consistent with previous studies reporting that parental input and home language environment in the second year of life are important for literacy skill development (Silvén et al., 2002; Suggate et al., 2018; Tamis-LeMonda et al., 2019), and extend previous studies relating parentese input to expressive language skills (Ramírez-Esparza et al., 2014, 2017a,2017b; Ferjan Ramírez et al., 2019, 2020). It has been suggested that parentese affects speech development because this unique speaking style increases infants’ perception of phonetic categories, and their ability to produce them (Kuhl et al., 1997; Cristià, 2011; Ramírez-Esparza et al., 2017a). The current study is the first to demonstrate a long-term effect of parentese input in infancy on emergent literacy skills at the age of 5-years.

The results of the behavioral analysis further indicate that Infants’ speech and speech-like vocalizations at 14-months predict letter-sound knowledge at age 5 years. Recent work has demonstrated that speech-sound production in pre-readers uniquely predicts word later identification in 2nd grade, with additional mediating effects of phonological awareness and letter-knowledge skills (Mues et al., 2021). However, it is not yet clear how different elements of speech-sound production and expressive language relate to emergent literacy skills before the onset of literacy instruction. While the current study suggests a relationship between infant speech and speech-like vocalizations at 14 months and letter-sound knowledge in 5-year-old preschoolers, it should be noted that no clear relationship was found between early child speech output and subsequent 5-year vocabulary. While this is somewhat surprising, it is also important to note that the current measure of child output captures the quantity of child speech, but not lexical diversity or sophistication. Therefore, it does not perfectly correspond to early vocabulary skill. It is also possible that environmental factors at later ages moderate the relationship between these variables over time.

Child speech at 6 and 24 months was negatively correlated with later reading measures, which was unexpected. Importantly, the child speech measure does not differentiate among categories of speech-like vocalization, such as canonical vs. non-canonical babbling. Children with lower 5-year vocabulary skills might therefore produce more, but less sophisticated, vocalizations at 6 and 24 months. Alternatively, child output at these ages might be less reliably measured, especially at 6 months, where children produce fewer vocalizations, overall. Notably, within the MRI sample, negative effects at 6 months were smaller, and non-significant. Future work, using a finer-grained manual coding of the LENA recordings at each age, will be needed to evaluate these possibilities. In contrast, at 14 months, children can be expected to produce canonical babbling (syllables produced with adult-like consonant vowel transitions), as well as a small number of early words. This time point may therefore contain greater individual variability related to expressive language development.

Finally, the results of the behavioral correlations indicate that parent-infant interactions, indexed by conversational turns at 14-months, predict letter-sounds knowledge at the age of 5-years. This effect was strongest for letter-sound knowledge in the subset of participants with MRI data. Importantly, the MRI group primarily included families who previously participated in a parental language intervention (Ferjan Ramírez et al., 2019, 2020), and this group had higher overall conversational turn counts, as compared to the larger sample. Future work is needed to clarify whether these findings generalize to a larger sample with a greater range of conversational exposure, and whether these results are specifically related to the environmental enrichment provided by the intervention.

The current study is the first to demonstrate long-term effects of parent-infant turn-taking on letter-sounds knowledge at the age of 5-years. However, we note that the effect was strongest in the subset of participants with MRI data, and therefore needs further examination in a larger sample. Importantly, the MRI group primarily included families who previously participated in a parental language intervention (Ferjan Ramírez et al., 2019, 2020), and this group had higher overall conversational turn counts, as compared to the larger sample. Future work is needed to clarify whether these findings generalize to a larger sample with a greater range of conversational exposure, and whether these results are specifically related to the environmental enrichment provided by the intervention.

Altogether, the behavioral results from the current study tie together the conclusions from previous studies and illuminate that early parental input, infants’ speech production, and parent-child interactions support not only language development, but the development of emergent literacy skills as well.

### Brain-behavior correlations

Infant vocalizations, parentese speech input, and parent-child conversational turns at 14-months correlated with estimates of myelin density within left AF 26-months. Parent-child conversational turns, but not infant vocalizations, also correlated with left SLF myelination at 26-months. No significant correlations with early language measures were found for the right hemisphere white matter tracts, or for ventral pathways (IFOF and ILF), reinforcing the idea that the emerging language network is left-hemisphere dominant very early in development. These results extend the findings from previous studies indicating that parent-child conversational turns and language skills in 4–6-year-olds correlate with concurrent structural connectivity in the left AF and SLF (Romeo et al., 2018b), storytelling related activation in the left inferior frontal gyrus (Romeo et al., 2018a), and structural plasticity in the superior marginal gyrus (Romeo et al., 2021).

Further, letter name and sound knowledge at 5-years correlated with estimated myelin density in the left arcuate and the left and right ILF at 26-months. These results extend previous studies indicating that brain structure and function in the left dorsal pathways in 5-year-olds predict later reading skills including letter-word identification (Wang et al., 2020; Yamasaki et al., 2021), phonological awareness (Yu et al., 2018), cross-modal audio-visual processing (Gullick and Booth, 2014, 2015), and word and pseudoword reading (Van Der Auwera et al., 2021). The current study indicates similar correlations between earlier left dorsal white matter structure and 5-year-olds’ letter names and sound knowledge, suggesting that these relations already exist in toddlers, long before the onset of reading instruction, and support reading acquisition.

Altogether, the results of the brain-behavior correlations from the current study indicate that the effects of early experience on the left dorsal pathways may have implications for later development of specific emergent literacy skills.

### Mediation analysis

Myelination of the left AF, estimated using quantitative MPF mapping at 26-months, was found to mediate the relationship between parent-child conversational turns and child speech at 14 months, and letter sound knowledge at 5 years. Myelin density estimates in the left arcuate were also found to mediate the relationship between parentese at 14 months and letter name knowledge at 5 years. These findings suggest a potential biological mechanism underpinning for the longitudinal relationship between parent-child interactions and later decoding skills.

The left AF and SLF have previously been found to be related to the development of expressive and receptive language skills (O’Muircheartaigh et al., 2014; Deoni et al., 2016; Salvan et al., 2017; Swanson et al., 2017; Romeo et al., 2018a,^2021^; Girault et al., 2019b; Sket et al., 2019), and emergent literacy skills (Lebel and Beaulieu, 2009; Yeatman et al., 2011; Saygin et al., 2013; Yu et al., 2018; Wang et al., 2020; Van Der Auwera et al., 2021; Yamasaki et al., 2021). Furthermore, previous studies have shown that the left AF correlates with parent-child conversational turns in 4–6-year-olds (Romeo et al., 2018a,b, 2021), and that left dorsal activation and plasticity mediate the relations between parent-child conversational turns and comprehensive language skills (Romeo et al., 2018a). Plasticity of the left dorsal structures has also been shown to mediate the relationship between intervention-related changes in parent-child conversational turns, and gains in comprehensive language skills (Romeo et al., 2021). The current results extend these studies and suggest that the left dorsal white matter, and specifically the AF, might serve as a mechanism by which language experience in infancy supports the development of subsequent emergent literacy skills at age of 5 years.

It has been shown that parent-child conversational turns and parentese enhance the infant’s speech and speech-like production, which in turn, encourage caregivers to respond and provide contingent feedback, and lead to a positive social feedback loop (Hirsh-Pasek et al., 2015; Gilkerson et al., 2018; Ferjan Ramírez et al., 2020; Romeo et al., 2021). The current data suggest that this process plays a role in the development of left dorsal language pathways, which in turn may facilitate emergent literacy skills. While the differing pattern for results for parentese vs. parent-child interaction hints that distinct developmental processes may be involved, future studies with additional neural measures and measurement time points are needed to clarify the contribution from specific components of early language input and experience.

### Limitations and future directions

There are number of limitations in the current study which need to be mentioned. First, the sample is limited to native English speakers and children without known environmental or genetic risk factors, such as lower SES or family history of dyslexia. Further, the sample includes many families who participated a parental language intervention from 6 to 18 months (16 out of the 20 MRI participants, and 38 out of the total 53 participants), which was previously found to increase parentese speech and parent-child conversational turn taking (Ferjan Ramírez et al., 2019, 2020). Finally, the MRI sample size is relatively small (*n* = 20). Future work is therefore needed to clarify whether the current findings generalize to a larger and more demographically diverse sample, with a greater range of conversational exposure, and whether these results are specifically related to the environmental enrichment provided by the intervention.

Future studies should examine how additional variables, such as lexical diversity in parent and child speech, relate to both emergent and longer-term literacy skills, and how measures of brain structure and function might relate to these effects. These goals can be achieved by larger longitudinal studies that follow participants from infancy to school-age using multiple approaches to measure brain structure and function, and behavioral language and literacy skills. Furthermore, as mentioned in the Introduction, according to SVR (Hoover and Gough, 1990), both decoding and language comprehension skills predict the ability to develop good reading skills. Hence, future studies should further investigate how language comprehension skills such as vocabulary, morpho-syntax, and narrative skills in pre-readers relate to parental input and child language early in life.

## Conclusion

In the current study we demonstrate for the first time a relationship between parental language input and parent-child interaction during late infancy and later emergent literacy skills in 5-year-olds, with an additional brain measure that suggests a biological mechanism for these effects, namely, developmental myelination of specific components of the left hemisphere’s emerging language network, the left dorsal pathways. The key findings are that first, Infants’ emergent speech production, together with parental use of parentese speech style and their conversational interactions with their infants directly predict emergent literacy skills, including letter names and sounds knowledge, phonological awareness, and expressive vocabulary in preschool. Second, Infants’ emergent speech production, together with parental use of parentese speech style and their conversational interactions with their infants directly relate to myelination of left dorsal pathways (specifically the AF, and SLF) at the age of 26-months. Third, emergent literacy skills, and specifically letter names and sounds knowledge in 5-year-olds directly relate to myelination of the left dorsal pathways (specifically the AF) at the age of 26-months. Fourth, left AF myelination at 26-months may account for the relationship between measures of emergent speech production, parental input, and parent-child interactions in infancy and letter name and sounds knowledge in 5-year-olds.

These findings contribute to our understanding of the brain mechanisms involved in reading development and break new ground by suggesting a potential mechanism by which language experience early in life scaffolds later reading acquisition. Further research is needed to test the mechanism hypothesis.

## Data availability statement

The raw data supporting the conclusions of this article will be made available by the authors, following reasonable request.

## Ethics statement

The studies involving human participants were reviewed and approved by The University of Washington Human Subjects Division IRB Committee. Written informed consent from the participants’ legal guardian/next of kin was not required to participate in this study in accordance with the national legislation and the institutional requirements.

## Author contributions

YW, EH, and PK contributed to the conception and design of the study. NF collected and analyzed the LENA data. NC and VY processed the quantitative MRI data to produce MPF maps. EH performed the diffusion MRI analysis. YW and EH organized the database, performed the statistical analysis, and wrote the first draft of the manuscript. All authors contributed to manuscript revision, read, and approved the submitted version.
